# The Influence of Maternal Cell Contamination on Fetal Aneuploidy Detection Using Chip-Based Digital PCR Testing

**DOI:** 10.3390/diagnostics11091607

**Published:** 2021-09-03

**Authors:** Anna Nykel, Rafał Woźniak, Agnieszka Gach

**Affiliations:** 1 Department of Genetics, Polish Mother’s Memorial Hospital Research Institute, 93-338 Lodz, Poland; 2 Chair of Statistics and Econometrics, Faculty of Economic Sciences, University of Warsaw, 00-241 Warsaw, Poland; rafal.wozniak@uw.edu.pl

**Keywords:** prenatal diagnostics, chip-based digital PCR, aneuploidy detection, maternal cell contamination, mosaicism

## Abstract

Prenatal samples obtained by amniocentesis or chorionic villus sampling are at risk of maternal cell contamination (MCC). In traditional prenatal analysis, MCC is recommended to be assayed by special tests, such as the short tandem repeat analysis and, if detected at a high level, may result in failed analysis report. The objective of this study was to test the ability of chip-based digital PCR to detect fetal aneuploidies in the presence of MCC. To determine the level of accuracy of MCC detection, an aneuploid male sample was subjected to serial dilution with an euploid female sample. DNA was extracted from prenatal samples and analyzed with QuantStudio 3D Digital PCR. Digital PCR analysis allowed the detection of trisomy 21, trisomy 18, and X monosomy accurately in samples with 90%, 85%, and 92% of MCC, respectively. Moreover, our results indicated that digital PCR was able to accurately confirm the presence of Y chromosome at up to 95% contamination. The amniotic fluid and chorionic villus sampling (CVS) received in our clinical laboratory was subjected to further analysis of MCC based on the aneuploidy assessment algorithm, resulting in the identification of 10 contaminated samples and four cases of true fetal mosaicism. We conclude that chip-based digital PCR analysis enables the detection of fetal aneuploidy with high levels of accuracy, even in cases of significant MCC. Importantly, the algorithm eliminates the need for maternal DNA and additional MCC tests, which reduces costs and simplifies the diagnostic procedure. The method is easy to set up and suitable for routine clinical practice.

## 1. Introduction

Prenatal samples obtained by amniocentesis or chorionic villus sampling (CVS) are at risk of contamination by maternal cells (MCC). The most common cause of MCC is the presence of maternal blood in amniotic fluid (AF) and maternal decidua in CVS. Compared to amniotic fluid, CVS is associated with a higher risk of MCC, as it is difficult to accurately remove the maternal decidua from the fetal cells. This risk of MCC is calculated as approximately 0.5% in AF and 1–2% in CVS [1]. Uncultured amniocytes present a high risk of MCC due to the presence of maternal blood in the cell pellet [2], while cultured CVS presents a high level of potential MCC associated with remaining maternal decidua [3].

The presence of maternal cells in prenatal samples is associated with a significant preanalytical risk of prenatal misdiagnosis. Such contamination is a less common problem in traditional cytogenetic analysis because short-term amniocyte and CVS cultures are rarely at risk of selectively growing out large quantities of maternal cells [4]. Determining the influence of contamination on the results is especially important when performing sensitive PCR-based molecular tests, where the presence of a small number of maternal cells can result in a positive result [5]. For this reason, several genetic societies, such as the American College of Medical Genetics, the UK Clinical Molecular Genetics Society, the Canadian College of Medical Geneticists, and Genomics and the Association for Molecular Pathology, recommend MCC analysis as the standard procedures for all prenatal samples undergoing molecular testing [6,7,8,9].

Digital PCR acts by partitioning the PCR reaction mixture into thousands of individual subreactions before amplification, following which the absolute level of the input DNA is quantified by counting the number of positive and negative PCR results, analyzing them based on the Poisson distribution [10,11]. According to the methods of separating reaction mixture, digital PCR could be classified into droplet-based digital PCR and chip-based digital PCR (cdPCR). Droplet dPCR creates microreaction units with water-in-oil and microfluidic technology [12]. Chip-based digital PCR is based on the base plate that is equipped with tens of thousands of microwells using microtechnologies [13]. Digital PCR based on endpoint analysis provides several advantages over the routinely used quantitative fluorescence PCR (QF-PCR), including absolute quantification without the need for a standard curve and higher accuracy [10,11,14].

The ability of chip-based digital PCR to detect mosaicism has been published in a proof-of-concept study, and it has been suggested that high levels of MCC may not affect the detection of fetal aneuploidy [15]. The purpose of this study was to determine the accuracy of chip-based dPCR for detecting fetal aneuploidy in the presence of MCC and the aneuploidy detection threshold for achieving accurate diagnostic results with MCC.

## 2. Materials and Methods

### 2.1. MCC Simulation

In order to determine the influence of MCC on the results of prenatal digital PCR testing, a dilution series was prepared in which a aneuploid male sample was mixed with various amounts of an euploid female sample to act as a contaminant (95%, 92%, 90%, 85%, 75%, 50% and 25% euploid sample). The DNA concentration of each sample was standardized to 10 ng/uL. Changes in Y chromosome copy number were then determined to detect MCC risks and the degree of contamination.

### 2.2. MCC Detection in Clinical Samples

Records for amniotic fluid and chorionic villus sampling received in our clinical laboratory for prenatal testing from September 2018 to April 2021 were reviewed for the presence of MCC during routine prenatal diagnostics. In accordance with the clinical algorithm adopted in our laboratory, each patient underwent a first-line test for rapid common aneuploidy detection using cdPCR while awaiting the result of full karyotype. Confirmation of cdPCR results was carried out using G-banded chromosome analysis. The aneuploidy detection was performed in response to a variety of indications, including advanced maternal age, abnormal biochemical results, fetal structural ultrasound abnormality, or family history of a genetic abnormality.

The study was conducted according to the guidelines of the Declaration of Helsinki and approved by the Bioethics Committee of the Polish Mother’s Memorial Hospital Research Institute (74/2018, issue date 11 September 2018). All pregnant women signed informed consents before the invasive procedure an additional consent to participate in a research study to analyze the most common aneuploidies using digital PCR method.

### 2.3. Chip-Based Digital PCR Workflow

Genomic DNA was extracted from uncultured CVS (approximately 1–5 mg) or AF (approximately 4–8 mL) using MagCore following proteinase K treatment and suspended in a final volume of 60 μL, according to the manufacturer’s protocol (RBC Bioscience, Taipei, Taiwan). The extracted DNA was measured by NanoDrop spectrophotometer (Life Technology, Carlsbad, CA, USA).

The digital PCR amplification was performed using a QuantStudio 3D Digital PCR System (Life Technology, Carlsbad, CA, USA) in a total reaction volume of 15 μL containing 7.5 μL QuantStudio 3D Digital PCR Master Mix (Life Technology, Carlsbad, CA, USA), 0.75 μL of the custom TaqMan Assays (Life Technology, Carlsbad, CA, USA), and 1 to 10 ng DNA template diluted in 6.75 μL. The primer and probe sequences and target combinations in duplex reactions are described elsewhere [16]. The reaction mixture was loaded onto a chip using QuantStudio 3D Digital PCR Chip Loader (Life Technology, Carlsbad, CA, USA). Thermal cycling was performed according to the manufacturer’s instructions: 96 °C for 10 min, 39 cycles of 60 °C for 2 min, 98 °C for 30 s, and a final extension at 60 °C for 2 min. The fluorescence signals of VIC and FAM were measured using QuantStudio 3D Digital PCR Instrument (Life Technology, Carlsbad, CA, USA).

### 2.4. Aneuploidy Analysis

Chip quality and raw data were analyzed using QuantStudio 3D AnalysisSuite Cloud v3.1.6 (Life Technology, Carlsbad, CA, USA). The concentration of the chromosome targets was determined based on positive and negative signals converted to copies/μL using Poisson statistics. The chromosome ratio was calculated by dividing the number of copies of chromosomes 21 and 18 and the X chromosome by that of the reference chromosome. To determine the aneuploidy, the z-score, i.e., the number of standard deviations from the mean of euploid sample datasets, was calculated as follows:

(1) $$z\text{-}\mathrm{score}=\frac{Ratio_{21:13}-mean\;Ratio_{euploid}}{SD\;Ratio_{euploid}}$$

where

(2) $$Ratio_{21:13}=\frac{\mathrm{Total}\;\mathrm{copy}\;\mathrm{number}\;\mathrm{of}\;\mathrm{chr}21}{\mathrm{Total}\;\mathrm{copy}\;\mathrm{number}\;\mathrm{of}\;\mathrm{chr}13}$$

The calculation example refers to the total number of FAM-labeled target on chromosome 21 (target chromosome) with respect to the reference total number of VIC-labeled targets on chromosome 13 (reference chromosome) obtained from the duplex reaction.

### 2.5. Statical Analysis

All statistical and data analyses were performed with the use of R statistical software v4.0.3 using FSA library v0.8.32 (R Foundation for Statistical Computing, Vienna, Austria). Correlation between variables was analyzed using the Pearson correlation coefficient (R^2^). The Mann–Whitney U-test was used for comparing z-scores across groups. Confidence intervals for specificity were calculated using the standard formula, while confidence intervals for specificity were obtained as proposed by Brown et al. [17]. *p*-values < 0.05 were considered statistically significant.

## 3. Results

### 3.1. MCC Simulation

**Figure 1:**
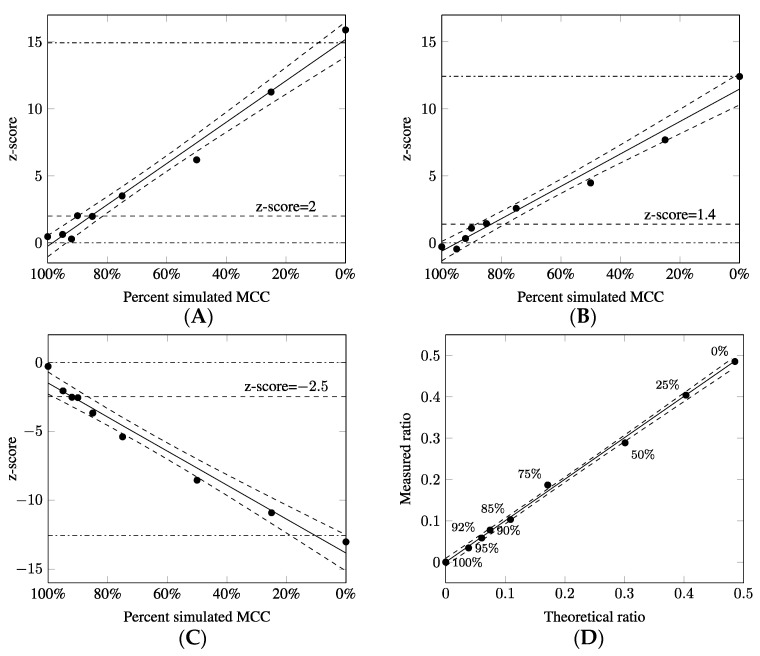
cdPCR results from the simulated MCC experiment. The z-score was plotted against percentage of simulated MCC for (**A**) chromosome 21, (**B**) chromosome 18, and (**C**) chromosome X. (**D**) The plots represent the relationship between measured and theoretical ratio for Y chromosome detection. The percentages indicate MCC level. The black line represents the linear regression; the circles represent the raw data, presented as the mean of two independent repeats. The dotted lines represent the 95% confidence interval. The dashed lines indicate the thresholds for the z-score. Dash-dotted lines represent the mean z-scores for euploid and aneuploid samples.

To determine the influence of MCC on the accuracy of aneuploidy detection using chip-based digital PCR, contamination was simulated using euploid female samples and aneuploid male samples. A set of serial dilutions was prepared to simulate 25% to 95% MCC. These were then subjected to analysis trisomy 21, trisomy 18, and X monosomy. The level of contamination was determined based on the detection of the Y chromosome in the mixture; this allowed the measured values to be correlated with the theoretical ones. To determine the aneuploidy of the test samples and confidence level of the results, the ratios and z-scores were calculated for chromosomes 21, 18 and Y. The cdPCR results are plotted in Figure 1.

Correlation coefficients (R^2^) were calculated for the dilution series to assess the relationships between the z-scores and the percentage contamination level. The calculated R^2^ values were found to be 0.9847 for the T21 series, 0.9796 for T18, and 0.9759 for X0 (Figure 1), indicating a strong correlation between measured aneuploidy content and MCC level. The R^2^ value for Y detection in the presence of MCC is 0.9981, indicating that experimental ratios were highly correlated with the theoretical ratios, as shown by linear regression fitting results.

In order to determine the thresholds for aneuploidy detection, the mean and standard deviation of the euploid and aneuploid samples were calculated using another 210 clinical samples (79 euploid female samples, 89 euploid male samples, 35 with T21, 7 with T18); all demonstrated over 200 copies/µL for targets on autosome chromosomes. The z-scores of the euploid samples for chromosomes 21, 18 and X were close to 0 ± 1 (range −2.588–2.800; −2.474–1.934; −2.926–2.270, respectively). In contrast, the z-scores of T21, T18, and X0 were 14.926 ± 2.166 (range 8.572–19.462), 12.422 ± 1.543 (range 11.335–15.835), and −12.579 ± 0.545 (range −14.174–(−11.341)), respectively. The difference in the z-scores in euploid and aneuploid group was significantly significant (*p*-value < 0.001 for T21, T18 and X0; Mann–Whitney U-test). To establish the thresholds for aneuploidy in the presence of MCC, sensitivity and specificity measurements were employed. 

The threshold for trisomy 21 was set to 2 with 100% specificity (100% CI, 98.199–100%) and 97.62% sensitivity (97.62% CI, 95.523–99.717%). The threshold for trisomy 18 was set to 1.4 with 100% specificity (100% CI, 95.801–100%) and 91.14% sensitivity (91.14% CI, 85.134–97.146%). The threshold for X monosomy was set to −2.5 with 100% specificity (100% CI, 97.949–100%) and 97.75% sensitivity (97.75% CI, 95.571–99.929%). The established thresholds for euploid and aneuploid group were applied to the serial dilutions of the simulated MCC (Figure 1). A subsequent analysis with these thresholds allows accurate identification of T21 in 90% of MCC, T18 in 85% of MCC, and X0 in 92% of MCC. 

**Figure 2:**
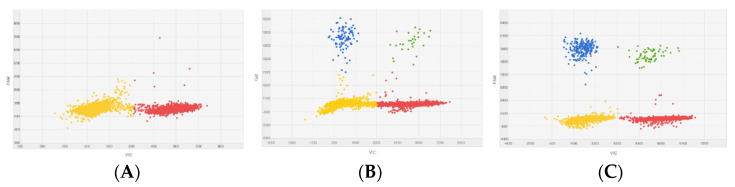
Scatterplots for Y chromosome detection in simulated MCC. Scatterplots representing fluorescence signals from probe targeting chromosome Y (FAM reporter dye) and probe targeting reference chromosome (VIC reporter dye) (**A**) detection of Y chromosome in 100% of simulated MCC, (**B**) detection of Y chromosome in 95% of simulated MCC, and (**C**) detection of Y chromosome in 92% of simulated MCC. The yellow dots represent wells without signal (no amplification), the green dots represent wells with FAM and VIC signal, the blue dots represent wells with a FAM signal, and the red dots represent wells with a VIC signal.

The aim of the next stage was to determine the detection threshold of the cdPCR method in the presence of MCC. For this purpose, simulated MCC experiments were performed based on the detection of the signal from the target from the Y chromosome compared to the target from the reference chromosome. Fluorescence signals from the Y chromosome at 100%, 95% and 92% simulated MCC are presented as scatterplots (Figure 2). These results indicate that cdPCR testing was able to accurately detect the Y chromosome at up to 95% MCC.

### 3.2. MCC Detection in Clinical Samples 

**Figure 3:**
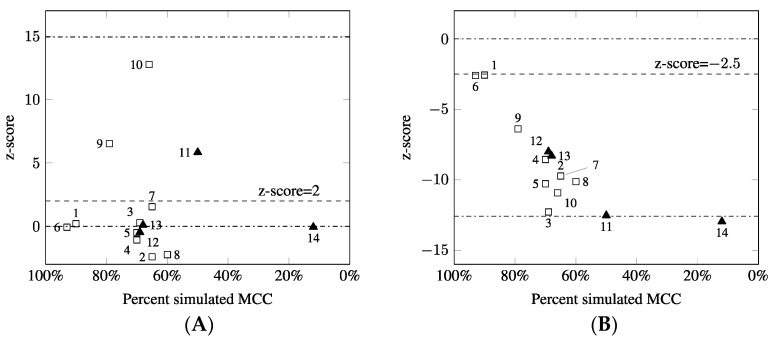
The results of MCC detection in clinical samples using cdPCR. The z-score was plotted against percentage of simulated MCC for (**A**) chromosome 21 and (**B**) chromosome X. For cases 11–14, the percentage shown relates to the mosaic pattern. The squares represent z-scores in clinical samples with MCC; the black triangles represent z-scores in clinical samples with true fetal mosaicism. Dashed lines indicate the thresholds for the z-scores. Dash-dotted lines represent the mean z-scores for euploid and aneuploid samples.

To determine the ploidy of the 21, 18 and X chromosomes, and the level of MCC based on the Y chromosome detection, the ratios, and z-scores were calculated for all clinical samples. The results of clinical samples obtained from dPCR testing are shown in Figure 3. The adopted clinical algorithm allowed for the identification of eight euploid male cases and two cases of T21 in the presence of MCC. In addition, one case of T21 mosaicism and three cases with mosaicism for monosomy X (one case of low-level mosaicism for a cell line with Y chromosome) were detected. 

In cases 1–10, samples demonstrating a positive Y chromosome signal together with X chromosome z-score values below the threshold for euploid samples indicate the presence of MCC. Determining the percentage of MCC was calculated according to the ratio of the Y chromosome to the reference chromosome (cases 1–10, MCC in the range of 60–93%). Euploid male samples were identified by a z-score value for the X chromosome in the range for euploid samples with simultaneous detection of the Y chromosome, while X monosomy was identified as the absence of the Y chromosome (cases 12, 13). A special issue is the identification of the Y-derived sequence in 45,X/46,XY because the detection of low signal chromosome Y may indicate contamination. However, the ratio of the X chromosome to the reference autosomal chromosome allows a single copy of the X chromosome to be identified (case 14). 

The aneuploidy for chromosomes 18 and 21 was determined for the clinical samples based on z-scores. Samples 10–13 were found to demonstrate trisomy 21 (z-score > 2), while remaining samples were classified as euploid (z-score < 2). The clinical samples demonstrated z-scores for chromosome 18 in the range of euploid samples (z-score < 1.4, data not shown). The adopted clinical algorithm allowed for the identification of all euploid and aneuploid samples despite the presence of MCC and to distinguish samples with contamination from samples with fetal mosaicism. The cdPCR results were consistent with the cytogenetic results (Table 1). Because samples 1 and 6 showed high levels of contamination (90% and 93%, respectively), the cdPCR results were reported as uninformative, with the caveat that fetal aneuploidy could be masked by MCC. 

## 4. Discussion

This is the first study to define the influence of maternal cell contamination on the interpretation of prenatal chip-based digital PCR analysis. Contamination with maternal cells may result in misinterpretation of diagnostic prenatal tests. As each prenatal test may have different accuracy in the presence of MCC, it is important to determine the exact threshold for detecting fetal aneuploidy in the presence of contamination. In addition, distinguishing between maternal cell contamination and true fetal mosaicism is a challenge for molecular testing.

Our results demonstrate that chip-based digital PCR is able to detect fetal aneuploidy in the presence of a high percentage of MCC. First, the threshold for the z-scores were established as 2 for trisomy 21 (100% of specificity, 97.62% of sensitivity), 1.4 for trisomy 18 (100% of specificity, 91.14% of sensitivity), and −2.5 for X monosomy (100% of specificity, 97.75% of sensitivity). These thresholds were then applied to the simulated MCC data. Digital PCR approach ensures the identification of fetal aneuploidy in the presence of high levels of MCC (90%, 85%, 92% of MCC in T21, T18, X0, respectively). Moreover, cdPCR is able to report a fluorescent signal from the Y chromosome as low as 5% of the fetal fraction, allowing the detection of MCC above 95%.

Chip-based digital PCR has proven to be a powerful first-line rapid test for aneuploidy detection in cases of MCC and mosaicism. In our clinical practice, 10 cases with MCC were identified at various levels (range 60–93%), eight were classified as euploid, and two as trisomy 21. In addition, four cases of true fetal mosaicism were observed (one case with trisomy 21, three cases with X monosomy, and one case with mosaic X0/XY). All results were consistent with the cytogenetic analysis. By determining the ratio of the X and Y chromosomes and the ratio of the X chromosome to the autosomal chromosome, cdPCR was able to distinguish samples with contamination from samples with a true mosaic pattern. Our results confirm that the applied algorithm eliminates the need to perform maternal DNA tests and additional MCC tests, which reduces costs and simplifies the diagnostic procedure. Our results demonstrate that QuantStudio 3D Digital PCR is an accurate method for fetal aneuploidy detection even in the presence of MCC and can be easily used as a rapid initial test for the most common aneuploidies in routine prenatal diagnostics.

The main limitation is the lack of unambiguous distinction between the maternal and fetal alleles, which may complicate the analysis. With a female fetus tested by cdPCR, it is not possible to conclusively determine whether the decreased z-score is due to contamination or mosaicism in the fetus. However, due to the high sensitivity of the method and the ability to perform an advanced analysis of all chromosomes simultaneously, it is often possible to identify samples with MCC.

The presence of a reduced ratio of X chromosome to the reference chromosome combined with a simultaneous increase in the ratio for chromosome 21 may indicate female trisomy 21 in the presence of MCC. Therefore, a clinical evaluation and a thorough analysis of the ratio of sex chromosomes and autosomal chromosomes is essential for the prenatal diagnosis. For female samples with z-scores below or above the threshold for normal or abnormal samples, additional MCC testing might be needed to verify the fetal origin of the results. As our data demonstrate that aneuploidies are detectable at 85–90% MCC, and the male result shows no variable ratio for the Y chromosome at MCC > 95%, there is a good chance that the cdPCR result excluded non-mosaic abnormalities without requiring additional MCC testing.

An additional limitation is the potential of MCC to mask low-level mosaicism. In such cases, as the effect of the MCC overlaps with that of mosaicism, the results need to be confirmed with those of another sample. However, in many cases, this change in value should not lead to a clinical misdiagnosis but only to the identification of a higher percentage of fetal mosaicism. However, a rare case of maternal X chromosome mosaicism, e.g., 45,X/46,XX, may interfere with the detection of fetal aneuploidy and may give the potential for false results in the presence of MCC. Moreover, our study is limited to assessing the influence of MCC for detection of 18, 21, X and Y chromosomes. Assessment of the influence of MCC for chromosome 13 requires further research.

Digital PCR operates by the simultaneous quantification of target nucleic acid using a large number of partitioned reactions. This approach provides several advantages over QF-PCR, including more precise measurements and absolute quantification of nucleic acids without standards. A comparison of digital PCR with other frequently used methods for rapid prenatal diagnosis, such as QF-PCR, fluorescent in-situ hybridization (FISH), and multiplex ligation-dependent probe amplification (MLPA), is presented in Table 2 [19,20,21]. However, unlike QF-PCR, cdPCR is not able to identify female triploidy and is less tolerant of samples with contamination. QF-PCR produces a characteristic pattern for samples with MCC based on the amplification of polymorphic microsatellite markers; however, this approach makes the results dependent on the presence of alleles in the population. In contrast, dPCR analyses the copy numbers of a single target, which is allele-independent. Another important advantage of cdPCR is its one-step protocol, which avoids the need for expensive equipment, such as a genetic analyzer, which enables the use of cdPCR in low-throughput laboratories.

Most molecular methods are able to detect low-level mosaicism. The accuracy of molecular tests in detecting more than one cell line largely depends on the level of mosaicism and the target chromosome. cdPCR is able to detect X monosomy in less than 10% compared to 20% in QF-PCR and MLPA [19,22]. The lowest detection threshold is demonstrated for the Y-derived sequences in 45,X/46,XY, which are detectable at a very low level, estimated as 5%, by cdPCR and QF-PCR [23].

Microfluidic dPCR has demonstrated much greater accuracy when detecting trisomy 21 compared to QF-PCR [16,24]. By comparing the amyloid protein sequences on chromosome 21 and the copy number of GAPDH on chromosome 12, digital PCR was able to detect trisomy 21 at a level of 10%. Elsewhere, dPCR has been found to demonstrate high sensitivity and specificity for aneuploidy detection, especially as part of non-invasive prenatal tests [25,26,27].

Our previous study detailed a novel approach for rapid aneuploidy detection using the QuantStudio 3D Digital PCR platform [18]. The results of the large-scale clinical evaluation demonstrated that cdPCR can detect aneuploidy with high sensitivity and specificity. In addition, previous research suggested that cdPCR can detect aneuploidy, even in cases of low-level mosaicism. However, this work was only preliminary, and further confirmatory studies are needed to determination of the detection threshold and the influence of contamination on the analysis. The ability of digital PCR to reliably detect fetal aneuploidy in the presence of MCC is not yet established.

Our research assesses the performance of QuantStudio 3D Digital PCR in detecting aneuploidy in the presence of MCC and determines the influence of MCC on the interpretation of results. Hence, a chip-based approach based on single target analysis is able to detect aneuploidy with a high level of accuracy, even in the presence of high levels of maternal cell contamination; it is also capable of distinguishing contamination from fetal mosaicism. Therefore, our solution demonstrates good clinical utility for prenatal diagnostics.

## Figures and Tables

**Table 1:** Detection of MCC in clinical experience.

Case No.	Sample Type	cdPCR Result			Follow-Up Cytogenetic Result
			z-Score for chr 21	z-Score for chr X	
1	AF	90% MCC, euploid male	0.198	−2.573	46,XY
2	AF	65% MCC, euploid male	−2.412	−9.739	46,XY
3	AF	69% MCC, euploid male	0.294	−12.285	46,XY
4	AF	70% MCC, euploid male	−1.076	−8.560	46,XY
5	AF	70% MCC, euploid male	−0.508	−10.280	46,XY
6	AF	93% MCC, euploid male	−0.069	−2.599	46,XY
7	AF	65% MCC, euploid male	1.550	−9.733	46,XY
8	AF	60% MCC, euploid male	−2.239	−10.129	46,XY
9	CVS	79% MCC, T21 male	6.519	−6.387	47,XY,+21
10	AF	66% MCC, T21 male	12.754	−10.926	47,XY,+21
11 ^1^	AF	50% mos, T21 male	5.838	−12.531	mos 47,XY,+21[13]/46,XY[10]
12 ^1^	CVS	69% mos, X0	−0.468	−7.996	mos 45,X[5]/46,XX[16]
13	CVS	68% mos, X0	0.102	−8.301	mos 45,X[10]/46,XX[14]
14	AF	12% mos, X0/XY	−0.059	−12.963	mos 45,X[28]/46,XY[6]

^1^ Two cases with identified mosaicism have been published previously [18]. CVS, chorionic villus sampling; AF, amniotic fluid; MCC, maternal cell contamination; mos, mosaicism; X0, X monosomy; T21, trisomy 21.

**Table 2:** Comparison of digital PCR and routinely used molecular methods for aneuploidy testing. Modified, based on previously provided data [19].

	cdPCR	QF-PCR	MLPA	FISH
Detection of aneuploidies of target chromosomes	+	+	+	+
Detection of mosaicism of target chromosomes	5–15%	5–20%	~20%	10%
Detection of MCC	+	+	+/−	+/−
Number of loci per chromosome	1	5/10	8 (13, 18, 21, X), Y (Y)	1
Sex chromosomes included	+	depends on kit	+	+
Detection of triploidy	male only	male, female	male only	male, female
Non-informative loci	−	+	−	−
DNA quality/concentration important	less than with QF-PCR, MLPA	less than with MLPA	yes	interphase nuclei needed
Results quality influenced by gestational age	no	no	no	yes
Workflow	only pre-PCR step	pre-PCR and post-PCR steps	pre-PCR and post-PCR steps, overnight hybridization,	labor-intensive, overnight hybridization
Equipment	QS3D Digital PCR system	thermal cycler, genetic analyzer	thermal cycler, genetic analyzer	fluorescent microscopy
Results time	<4 h	<6 h	<48 h	<24 h

QF-PCR, quantitative fluorescent polymerase chain reaction; MLPA, multiplex ligation-dependent probe amplification; FISH, fluorescence in-situ hybridization; QS3D Digital PCR system, QuantStudio 3D Digital PCR system.

## Data Availability

The raw data from QuantStudio 3D Digital PCR AnalysisSuite Cloud documenting the number of copies of the analyzed chromosomes and chip quality data from clinical samples and simulated MCC samples study are available from the corresponding author.
